# Neurosarcoidosis resulting in thoracic radiculopathy: a case report

**DOI:** 10.1186/s13256-019-2065-0

**Published:** 2019-05-05

**Authors:** Hayam Hamodat, Allen Tran

**Affiliations:** 1 Department of Medical Neuroscience, Dalhousie University, Room 13-B1, 5850 College Street, PO Box 15000, Halifax, NS B3H 4R2 Canada; 2 Department of Medicine, Dalhousie University, Room 409 Bethune Building, 1276 South Park Street, Halifax, NS B3H 2Y9 Canada

**Keywords:** Thoracic radiculopathy, Neurosarcoidosis, Sarcoidosis, Immunosuppression

## Abstract

**Background:**

Thoracic radiculopathy is a rare etiology of vague abdominal pain. There are few reports of neurosarcoidosis manifesting as a thoracic radiculopathy, which highlights the diagnostic challenge.

**Case presentation:**

A 54-year-old Caucasian man was being investigated for right upper quadrant abdominal pain and was found to have diffuse lymphadenopathy on imaging. He was eventually diagnosed with sarcoidosis. Over time, his abdominal pain progressed to neuropathic pain along his T7–T11 dermatome. Magnetic resonance imaging revealed findings consistent with a thoracic radiculopathy due to neurosarcoidosis. The patient received corticosteroids for treatment of neurosarcoidosis and immunosuppressant therapy to manage neuropathic pain.

**Conclusions:**

This case report highlights the importance of considering thoracic radiculopathies in the differential diagnosis of vague abdominal pain and explores guidelines in diagnosing neurosarcoidosis in the absence of neural tissue biopsy.

## Background

Sarcoidosis is a systemic, multiorgan disease characterized by the histological evidence of noncaseating epithelioid cell granulomas [1]. Neurologic manifestations occur in 5–10% of individuals with sarcoidosis [2–5] and are the presenting symptom in 50% of patients who are eventually found to have neurosarcoidosis [2]. The two most common presentations of neurosarcoidosis are cranial neuropathies (specifically, facial nerve palsy) and meningitis. However, patients may present with various symptoms depending on the site of granuloma formation [2, 6].

Sarcoidosis of the spinal cord and nerve roots is relatively rare [7]. One case series found spinal cord involvement in 0.43% of patients with sarcoidosis [8]. However, the same review also reported an autopsy series that suggested spinal cord involvement was as high as 20% in patients with sarcoidosis. In a case series of 37 patients with suspected or confirmed sarcoid involvement of the spinal cord, 13 had intramedullary lesions, 16 had extramedullary lesions, 4 had both intra- and extramedullary lesions, and 4 had macroscopically normal spinal cords [8]. There are few documented cases of neurosarcoidosis causing radiculopathy [9–13]. Mechanisms of spinal root nerve pain in sarcoidosis may include direct compression of the root, ischemic changes of nerves due to granulomas, or inflammatory mechanisms [10].

We present a case of an uncommon etiology of abdominal pain that highlights the need to consider thoracic radiculopathies in cases of undiagnosed abdominal pain. This case also highlights a rare presentation of neurosarcoidosis as a thoracic radiculopathy. We discuss guidelines for diagnosing neurosarcoidosis in the absence of neural tissue biopsy and potential treatment regimens for these patients.

## Case presentation

A 54-year-old Caucasian man was referred to our hospital for management of sarcoidosis. This was found in investigations for a 2-year history of progressive right upper quadrant abdominal pain. He underwent computed tomography (CT) of his abdomen and pelvis, which revealed significant findings of diffuse lymphadenopathy. CT of his chest demonstrated bilateral hilar and mediastinal lymphadenopathy concerning for lymphoma. The patient underwent thoracic lymph node biopsy, which revealed noncaseating granulomas, consistent with sarcoidosis.

His abdominal pain was initially occurring every few days and was sharp in nature. It was located along the right costal margin. His pain progressed to daily episodes and eventually constant discomfort. The pain began to radiate around to his back along the tenth rib. Over the course of 1 year, his pain became neuropathic with symptoms of allodynia and intermittent, shock-like pain. His pain also migrated to his T7–T11 dermatomes. He had no other symptoms.

The patient’s past medical history was significant for an 8-year history of type 2 diabetes mellitus without any known complications, as well as dyslipidemia, asthma, osteoarthritis, bilateral knee replacements, and a cholecystectomy. His medications at the initial visit included metformin, gliclazide, liraglutide, hydrochlorothiazide, trandolapril, aspirin, celecoxib, tramadol/acetaminophen, atorvastatin, and inhaled budesonide/formoterol. His family history included premature cardiovascular disease in both of his parents. One brother had lung cancer and his sister had ovarian cancer. He is a lifelong nonsmoker and consumes alcohol socially about once per month. He is a medical administrator with the military and had traveled extensively in the past with the military but had only visited resorts in Central America over the last 2 years. He has a parrot at home and no other animal contacts.

On initial examination, his blood pressure was 120/76 mmHg, and his heart rate was 89 beats/min and regular. He had no jugular venous distention. His heart sounds were normal with no extra sounds or murmurs. His lungs were clear to auscultation. His abdomen was soft but mildly tender on palpation in the right upper quadrant. His liver edge was palpable about 2 cm below the costal margin and was smooth. He did not have any signs of splenomegaly on examination. His extraocular movements were normal, as were the results of the rest of his cranial nerve testing. His muscle bulk and tone were normal. His strength was 5/5 on Medical Research Council grading throughout. His reflexes were 2+ bilaterally. He had no evidence of ataxia, and his gait was normal. His plantar reflexes were downgoing bilaterally, and no pronator drift was present. His sensation was normal initially, but on serial examinations over the next year, he developed allodynia over the right-sided T7–T11 dermatomes with pain to light touch. He also had decreased sensation to pinprick and light touch in this area.

Biochemical and hematologic serum test results were within normal limits (Table 1). His C-reactive protein was normal at 4.70 mg/L. His 24-h urine calcium was elevated at 12.5 mmol/24 h (normal range, 0.0–7.4 mmol/24 h). His electrocardiogram (ECG) was normal. Results of his pulmonary function testing were normal with a forced expiratory volume in 1 second (FEV_1_)/forced vital capacity (FVC) ratio of 76%, FEV_1_ 89% predicted, FVC 93% predicted, total lung capacity 99% predicted, and diffusing capacity of the lungs for carbon monoxide 125% predicted.

**Table 1:** Relevant laboratory test results obtained during investigation of patient’s abdominal pain

Test	Results
White blood cell count (4.5–11.0 × 10^9^/L)	9.32 × 10^9^/L
Hemoglobin (120–180 g/L)	176 g/L
Mean corpuscular volume (80.0–97.0 fl)	89.7 fl
Platelet count (150–350 × 10^9^/L)	207 × 10^9^/L
Sodium (136–145 mmol/L)	141 mmol/L
Potassium (3.4–5.0 mmol/L)	3.8 mmol/L
Chloride (100–110 mmol/L)	102 mmol/L
Calcium (2.20–2.60 mmol/L)	2.50 mmol/L
Phosphorus (0.74–1.52 mmol/L)	0.94 mmol/L
Magnesium (0.66–1.07 mmol/L)	0.81 mmol/L
Creatinine (49–90 μmol/L)	72 μmol/L
Alanine aminotransferase (0–45 U/L)	45 U/L
Aspartate aminotransferase (5–45 U/L)	28 U/L
Alkaline phosphatase (38–150 U/L)	59 U/L
Total bilirubin (0.0–20.4 μmol/L)	13.8 μmol/L
Albumin (35–50 g/L)	42 g/L
Thyroid stimulating hormone (0.35–4.30 mIU/L)	0.88 mIU/L
Hemoglobin A1c (4.6–6.3%)	6.3%

**Fig. 1:**
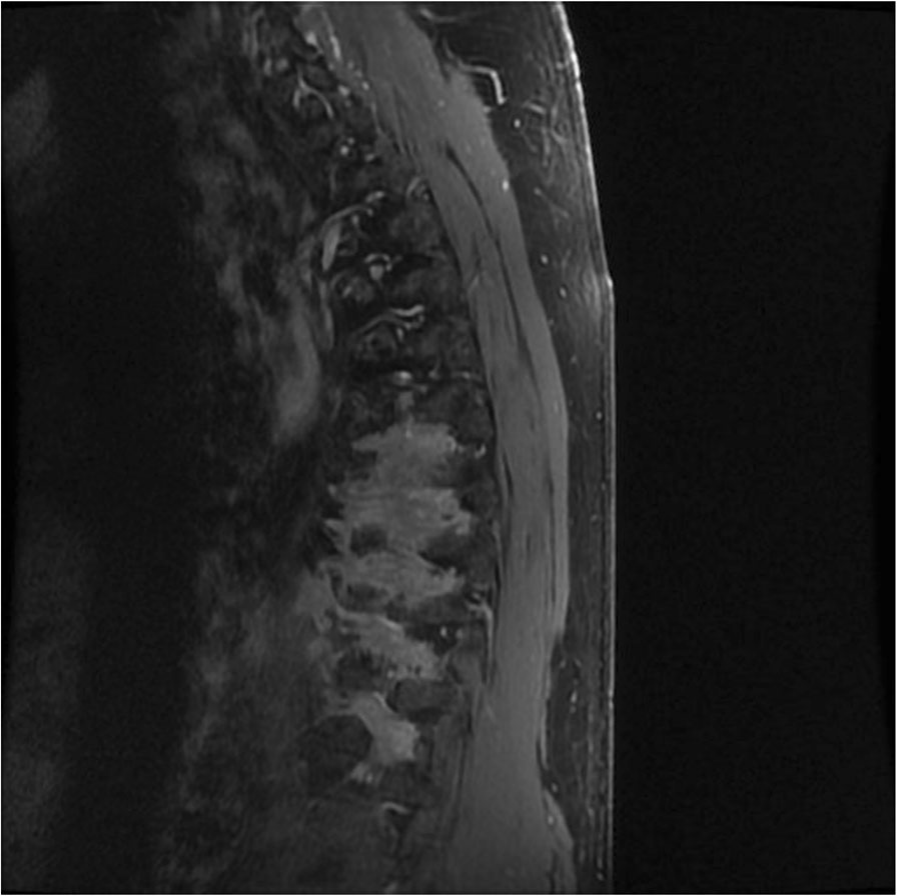
Sagittal magnetic resonance imaging of the thoracic spine. T1-weighted image with gadolinium enhancement displaying abnormal right paravertebral soft tissue thickening and enhancement along multiple thoracic levels

**Fig. 2:**
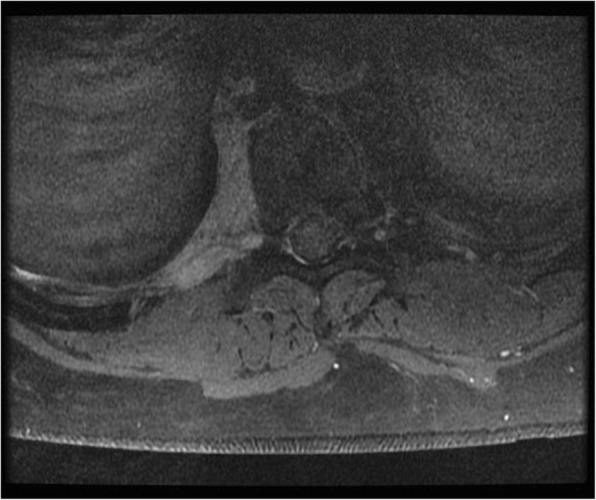
Transverse magnetic resonance imaging of the thoracic spine. T1-weighted image with gadolinium enhancement displaying abnormal paravertebral soft tissue thickening and enhancement of the right nerve root entering the neural foramina

Magnetic resonance imaging (MRI) of his spine revealed abnormal thickening and enhancement of paravertebral soft tissues along the right intercostal spaces that extended from T7 to T12 (Figs. 1 and 2). There was enhancement of the right thoracic nerve roots from T5 to T10. This was in keeping with neurosarcoidosis. MRI of the patient’s head did not reveal any evidence of sarcoidosis involving his brain. Cerebrospinal fluid (CSF) analysis revealed 3 × 10^6^ white blood cells/L (100% lymphocytes), 1 × 10^6^ red blood cells/L, total protein 0.47 g/L, and glucose 6.65 mmol/L.

After MRI of the patient’s spine, he was started on prednisone 75 mg daily. Gabapentin was initiated. There was minimal change in his symptoms after 3 weeks. Azathioprine was added (titrated up to 225 mg daily) over the next 6 months while prednisone was tapered. His 24-h urine calcium normalized, but he did not experience significant changes in his neuropathic pain. Mycophenolate mofetil and infliximab (every 8 weeks) were then initiated, and azathioprine was stopped. However, after four doses of infliximab, he noticed an overall decrease in his neuropathic pain.

## Discussion

In this case report, we share a presentation of vague abdominal pain that was diagnosed as neurosarcoidosis causing thoracic radiculopathy on the basis of imaging and thoracic lymph node biopsy. There are few reported cases of neurosarcoidosis causing radiculopathy, and specifically, involvement of the thoracic spine is rare [9–13]. The particularity of this case report is the presentation of abdominal pain associated with the thoracic spine pathology, which, to our knowledge, has not been reported previously.

Neurosarcoidosis should be suspected in individuals with systemic manifestations of sarcoidosis who develop new neurologic manifestations [6]. In individuals presenting with relatively uncommon neurologic symptoms that are highly prevalent in neurosarcoidosis, such as uveoparotid syndrome and cranial oligoneuropathy, or in those with neuroinflammatory diseases where more common diagnoses have been excluded, should also have neurosarcoidosis considered [14]. The most widely accepted diagnostic criteria, published by Zajicek and colleagues [15], classify neurosarcoidosis as definite, with nervous tissue biopsy confirmation, or probable, when neurologic inflammation is evident in the context of systemic sarcoidosis with the exclusion of alternative diagnoses. Possible neurosarcoidosis is considered when: symptoms are typical of neurosarcoidosis, the criteria for definite or probable neurosarcoidosis are not met, and other etiologies of the neurologic manifestations have been excluded.

Though a neural tissue biopsy is preferred to achieve a definite diagnosis, this is commonly too invasive and not practical. A probable diagnosis of neurosarcoidosis can be made using evidence of central nervous system inflammation based on MRI or CSF, such as elevated protein, pleocytosis, increased immunoglobulin G indices, or presence of oligoclonal bands [6, 15]. Individuals with suspected neurosarcoidosis and no previous history of systemic disease should be evaluated for other organ manifestations. Commonly, this includes a chest radiograph, ECG, routine blood tests including liver testing and calcium levels, urine calcium, CT of the abdomen and pelvis, pulmonary function tests with diffusion study, and ophthalmic evaluation with slit-lamp examination [6]. CSF examination should include cultures, cytology, and flow cytometry to rule out infectious and malignant causes in the differential diagnosis. Investigations to rule out other causes of granulomatous disease, such as tuberculosis and vasculitis, should be considered.

Particularly when nerve tissue biopsy is not a practical option for definitively diagnosing neurosarcoidosis, neural manifestations of sarcoid disease can mimic many other etiologies. For example, individuals presenting with neural involvement of the optic nerve, spinal cord, or brainstem may need to be investigated for multiple sclerosis [16]. Facial nerve palsies and nerve root irritation may be associated with Lyme disease. Individuals with meningeal involvement may need to be examined for vasculitic disease such as granulomatosis with polyangiitis or Behçet’s disease. Depending on the localization of neurosarcoidosis, investigations for differential diagnoses may differ.

Few case reports have described isolated involvement of thoracic spine roots in neurosarcoidosis, and the presentation varies between cases. In one case report of bilateral T6 level radiculopathy, the patient experienced anterior chest discomfort with hypesthesia and hypalgesia with preserved sensation of the medial region of the back [12]. In a case report of T4–T6 involvement, the patient presented with left-sided chest pain that radiated to the back and hypalgesia in the bilateral T3–T10 dermatomes [10]. The majority of cases of spinal nerve root neurosarcoidosis involve multiple nerve roots and bilateral lesions [15].

Regardless of the underlying etiology, thoracic radiculopathy is often unrecognized because symptoms of chest pain or vague abdominal pain may represent a myriad of conditions [16]. Physical examination of thoracic radiculopathies poses another challenge, because a bandlike distribution of sensory changes may not be clear, and there is no reliable physical examination maneuver for muscle weakness in the thorax or abdomen. Electromyography of the thoracic paraspinal, intercostal, or abdominal muscles may be done [13]; however, these are not easily accessible muscles. Many cases of thoracic radiculopathy result in excessive imaging and laboratory testing; however, with a broad differential diagnosis, thoracic radiculopathy rests largely as a diagnosis of exclusion [16].

There are no consensus guidelines or clinical trials on the treatment of neurosarcoidosis, owing to the rare occurrence of this disease. First-line therapy for many patients is corticosteroids. On the basis of anecdotal evidence, clinical remission occurs in up to 70% of patients receiving an optimal immunosuppressive regimen [17]. Some patients receive a combination of prednisone with immunosuppressive treatments, including methotrexate, chloroquine, hydroxychloroquine, cyclophosphamide, and infliximab [7]. In patients with neurosarcoidosis affecting the spinal cord, 65.9% showed clinical improvement, 24.6% deteriorated, and 9.5% remained stable. However, many patients with initial remarkable improvement with steroid therapy later had recurrence of symptoms [18].

## Conclusions

Our case report highlights the diagnostic challenge of neurosarcoidosis and the high index of suspicion necessary for detection of thoracic radiculopathies in particular. There is no high-quality evidence to provide guidance regarding management of this rare condition.

## Abbreviations

CSF: Cerebrospinal fluid
CT: Computed tomography
ECG: Electrocardiogram
FEV_1_: Forced expiratory volume in 1 second
FVC: Forced vital capacity
MRI: Magnetic resonance imaging
